# Manual of Anatomy

**Published:** 1907-09

**Authors:** 


### Reviews of Books.

**Manual of Anatomy.** By A. M. Buchanan, M.A., M.D. Vol. I. Pp. xvi., 596. London: Baillière, Tindall and Cox. 1906.

The student who is anxious to avoid knowing too much—and he is not uncommon—will welcome this book, because it seems small, and not overburdened with facts. It is a complete surprise to find that so much information has been conveyed in so small a volume. The matter has been well selected, the diction is terse and simple, the numerous illustrations are quite as good as in any English text-book, and one cannot help thinking that this book will prove a serious rival to the much more bulky works, such as Gray, Morris, &c., and we shall indeed be surprised if a successful career does not await this book, if the second volume be as good as the first, the student will welcome a volume of so convenient a size which at the same time contains such a fund of information.

It is, however, unlikely that in a first edition such a book should be absolutely free from blemishes, and there are a few to which we think well to draw the attention of the author.

The account of the development of the chondro-cranium is that which is found in all the text-books. It is not only antiquated, but absolutely erroneous. It has long been pointed out that the notochord has a quite different relation to the chondrocranium from that represented here; it is also well-known that the terms "parachordal" and "trabeculæ" have no real warrant in the human skull. The accounts of the processes of ossification of the various bones will bear revision, such as those of the malar bone, the mandible, the upper jaw, the sphenoid, the seventh cervical vertebra, the sacrum, the coracoid process of the scapula and ribs, and maybe others.

Speaking generally, the general description of the bones is good, and most of the illustrations are of high excellence, and a credit to the artist. Fig. 84, though very good, is too small; and Fig. 65 does not quite correspond with the text, for although the "nasal groove" on the posterior surface of the nasal bone is shown correctly running down to the middle of the lower margin of the nasal bone, and is said in the text opposite to contain the nasal nerve, yet a nasal notch quite unconnected with this groove is said to convey outwards the terminal cutaneous branch of the nasal nerve. In the description of the cervical vertebræ, the costal and transverse processes are carefully described and distinguished from one another, yet in the description of the seventh cervical vertebra both are alluded to as transverse processes, and the old terms "anterior and posterior tubercles" are used.

With regard to the thoracic vertebræ, more might have been made of the distinguishing characters of the eleventh vertebra, especially worthy of mention being the fact that the inferior articular processes, or if not they, small tubercles at the root of the spine project below the plane of the body and spine, which never happens in the tenth or those thoracic vertebræ above, but always in the twelfth and all below. That very characteristic feature of the fifth lumbar vertebra, viz. the springing of the costal process from the body, might have been made more of, as well as the fact that the transverse diameter of the neural arch greatly exceeds its vertical.

With regard to the long bones, one notices the old fiction repeated, "that during fœtal life the fibula articulates with the femur;" and the same error with reference to the attachment of the pronator teres to the radius is repeated.

One notices that the structure of the calcaneum is not quite intelligible as rendered here, and the area of attachment of the inner head of the accessorius is surely much too small.

The sections on muscles and arteries and nerves of the limbs, which complete the book, leave little to find fault with; but one feels bound to call attention to the scanty description of the articular branches of the external popliteal nerve.

With the exception of the blemishes to which we have drawn attention, and which no doubt will be remedied in editions which we feel sure will follow, Volume I. is excellent, and we strongly recommend it to the notice of the student who is not doing the most advanced work. It is a creditable addition to the already large number of students' text-books of anatomy—creditable alike to author, artist and publisher.

